# Enhanced Wear Resistance and Thermal Dissipation of Copper–Graphene Composite Coatings via Pulsed Electrodeposition for Circuit Breaker Applications

**DOI:** 10.3390/ma17236017

**Published:** 2024-12-09

**Authors:** Daniele Almonti, Daniel Salvi, Nadia Ucciardello, Silvia Vesco

**Affiliations:** Department of Enterprise Engineering “Mario Lucertini”, University of Rome Tor Vergata, 00133 Rome, Italy; daniel.salvi@uniroma2.it (D.S.); nadia.ucciardello@uniroma2.it (N.U.); silvia.vesco@uniroma2.it (S.V.)

**Keywords:** composite coating, ANOVA, tribology, electrodeposition, graphene nanoplatelets

## Abstract

Copper, though highly conductive, requires improved wear resistance and thermal dissipation in applications that involve continuous movement and current-induced vibrations, such as power breakers. Conventional solutions, such as copper–tungsten alloys or lubricant use, face limitations in durability, friction, or environmental impact. This study explores the development of copper–graphene (Cu-GNPs) composite coatings using pulsed electrodeposition to enhance the tribological, thermal, and mechanical properties of circuit breaker components by adopting an industrially scalable technique. The influence of deposition bath temperature, duty cycle, and frequency on coating morphology, hardness, wear resistance, and heat dissipation was systematically evaluated using a 2^3^ full factorial design and an Analysis of Variance (ANOVA). The results revealed that optimized pulsed electrodeposition significantly improved coating performance: hardness increased by 76%, wear volume decreased by more than 99%, and friction coefficient stabilized at 0.2, reflecting effective graphene integration. The addition of graphene further improved thermal diffusivity by 19.5%, supporting superior heat dissipation. These findings suggest that pulsed copper–graphene composite coatings offer a promising alternative to traditional copper alloys, enhancing the lifespan and reliability of electronic components through improved wear resistance, lower friction, and superior heat transfer.

## 1. Introduction

Copper is currently the most widely used material in electronics in both the distribution and transformation of electrical energy, because of its excellent conducting properties. Among the various applications of copper in electronics, the implementation of power switchers is of scientific interest. In fact, these components require various precautions to perform their best and increase their long-term reliability [1]. In particular, they are subjected to a normal load to ensure their contact when closed, and at the same time, they experience a reciprocal linear movement for their opening and closing [2], in addition to a constant vibration during use due to the passage of current that leads to fretting-type wear [3]. Low friction is required to allow the switcher to slide properly when in use. In addition, under defined operating conditions, low friction would reduce the wear of the component, extending its service life [4], which in the case of pure copper is significantly pronounced [5]. Castaños et al. [6] studied the impact of different lubricants on the wear of industrial circuit breakers by varying viscosity and thickeners and showed an improvement in tribological properties as the viscosity of the grease increased. Drawbacks due to lubricant usage are mainly represented by the necessity of routine maintenance to keep the contact lubricated and a loss of electrical contact in the case of excess lubricant. A route to improve the wear resistance of the components in question is the use of highly hard alloys such as copper–tungsten alloys [7] that, because of the tungsten, show remarkable wear resistance but maintain high friction. Zhang et al. studied the impact of tungsten particle size on component sintering and subsequent wear resistance, showing optimal performance for powders with a size of 400 nm [8]. However, the use of nanosized powders restricts industrial use due to various risks such as explosion during storage and handling [9]. Wang et al. studied the optimal percentage of tungsten in Cu-W alloys for wear reduction, with the best tribological conditions obtained from scenarios with 70 or 80 wt% tungsten [10]. Despite their high wear resistance, the studied alloy still features a coefficient of friction greater than 0.3. A further improvement in the wear performance of the Cu-W alloy has been achieved through the introduction of alumina particles [11], which, on the other hand, decrease the electrical properties.

A widely studied alternative is the introduction of other elements in the alloy, such as chromium [12] or zirconium [13], favoring wear resistance, which increase the production costs, as well as the use of silver–tungsten alloys [14], where the presence of tungsten is mandatory because of the weak wear resistance properties of silver. A more economical and easily industrialized alternative is the use of metal or composite coatings to help extend contact life [15]. Segura-Cárdenas et al. proposed a zinc coating to improve wear performance while maintaining good electrical properties. The electrodeposition of zinc requires sulphuric acid in the deposition bath and a layer of copper to promote the adhesion to the substrate [16]. Alternatively, the use of tin coatings has been evaluated in comparison with silver coatings, which enhances heat dissipation and corrosion resistance but not tribological performances [17] because of the poor mechanical properties of tin.

As an alternative to metal coatings, a material with outstanding mechanical and physical properties, such as graphene, can represent a great step forward in the realization of electronic components [18]. The use of graphene–copper composites in the manufacturing of power switches led to an improvement in the hardness and electrical conductivity of the component. Nevertheless, powder sintering technology, applied to implement the composite, is expensive and hardly manageable [19]. Graphene and its derivatives, such as graphene nanoplatelets (GNPs), have also been studied as reinforcements of metal–matrix composite coatings to improve tribological performance [20]. In particular, electrodeposited nickel–graphene coatings have been extensively studied for improved wear [21] and corrosion resistance [22]. Nickel coatings, however, are particularly expensive and environmentally unsustainable due to the toxicity of the deposition bath [23,24]. The use of copper–graphene composite coatings, on the other hand, appears to be less explored in the literature. Cui et al. proposed a Cu-GNP composite coating for ultrahigh voltage circuit breakers that exhibited a 25% improvement in microhardness compared to the bare copper substrate, also significantly reducing the coefficient of friction [25]. A direct current approach was used, while pulse current electrodeposition could lead to improved mechanical properties of the coating, as previously explored for neat copper [26] and tin–copper alloy [27] coatings.

In this work, the impact of the main parameters for pulsed electrodeposition was evaluated for the development of functional copper–graphene coatings, investigating for the first time the role of pulse parameters in the manufacturing of Cu-GNPs composite coating for circuit breakers. Specifically, through the implementation of a complete 2^3^ factorial plan, the deposition bath temperature, duty cycle, and wave function frequency were varied simultaneously. The main application of the Cu-GNP coatings examined is to obtain high-performance circuit breakers, so tribological, thermal, and adhesion evaluation tests were carried out. All process parameters studied show a significant impact, confirmed by Analysis of Variance (ANOVA), on the performance of composite coatings. In particular, a maximum hardness increment of 76% was observed, which led to a wear volume reduction of more than 99% with an average friction coefficient of 0.2. Additionally, pulsed copper–graphene deposition led to a 19.5% increase in thermal diffusivity.

## 2. Materials and Methods

### 2.1. Materials and Fabrication

The objective of the proposed work is to evaluate the thermal and tribological properties of copper–graphene coatings made by electrodeposition on copper substrates. Pure copper sheets (99.9 wt% copper, B.L. Sistemi s.r.l., Rome, Italy) with a size of 40 × 20 × 2 mm were used as substrates for experimentation. Graphene copper depositions were conducted with an average current density of 2 A/dm^2^ to achieve a theoretical thickness of 25 µm, determined by Faraday’s law:

(1) $$Theorical\;thickness\left[µm\right]=\frac{t\cdot i\cdot M}{F\cdot \nu \cdot d}$$

where *t* is the deposition time in seconds, *i* is the average current density (in A/cm^2^), *M* is the molar mass of copper ions (in g/mol), *F* is the Faraday constant (96,485.3321 s·A/mol), ν is the number of valence electrons of copper (equal to 2), and *d* its density of copper (in g/cm^3^).

In order to have a clean and oxide-free surface, before deposition, an electrochemical cleaning treatment was applied to the copper substrates. A two-step chemical degreasing and electrochemical pickling industrial process, followed by a rinse in deionized water, was used. In particular, the degreasing treatment consisted of a 3 min immersion in a degreasing solution (Condorine 156, Condoroil Chemical s.r.l., Casale Litta, Italy) with an applied cathodic current density of 8 A/dm^2^, while the pickling step consisted of a 2 min immersion in a pickling water-based solution (744P, Condoroil Chemical s.r.l., Italy).

**Figure 1:**
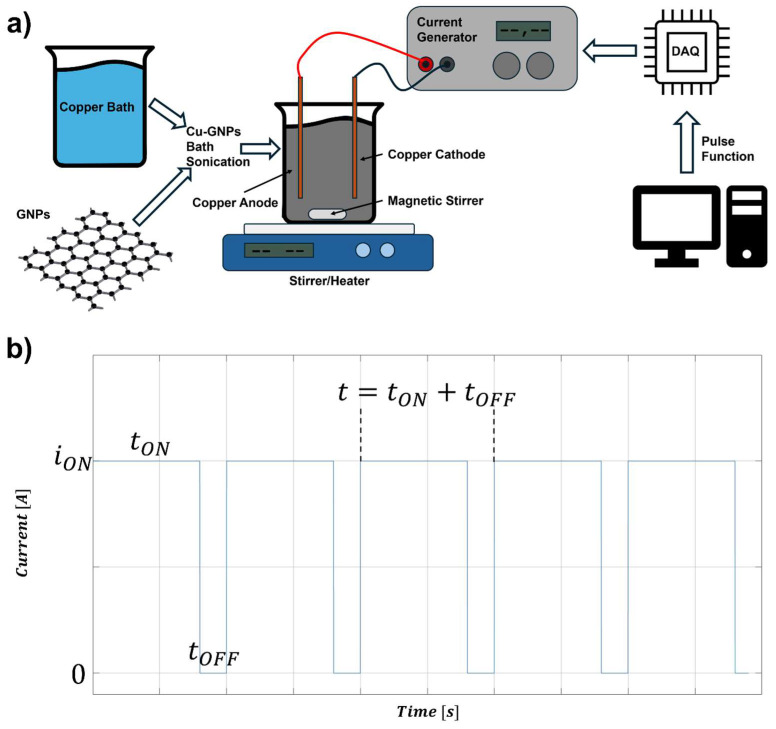
(**a**) Experimental set-up for the electrodeposition; (**b**) pulsed wave function.

As reported in Figure 1a, the GNPs-Cu deposition bath was obtained by adding GNPs (G2, Nanesa s.r.l., Rome, Italy) with the specifications in Table 1 to a sulfate-based copper bath (Table 2); a small amount of copper chloride was needed to help with the electrical conductivity of the bath, without giving extra copper supply, as suggested by [28]. Subsequently, the bath was sonicated for 20 min with a Sonic Materials VCX 750 (Thermo Fisher Scientific Inc., Waltham, MA, USA) instrument to ensure the correct dispersion of GNPs flakes in the solution. Pulsed electrodeposition was performed using an AC source (BOP 50-2D, KEPCO, Naju, Republic of Korea), controlled through a Data Acquisition System (DAQ, National Instruments, Austin, TX, USA) and LabView 2024 Q2 software, generating a square wave function controlling duty cycle and frequency. All the deposition took place in a 200 mL solution constantly stirred at 300 rpm by means of a magnetic stirrer varying the bath temperature, using a copper plate as the sacrificial anode. The experiment consisted of a 2^3^ full factorial plane, taking into account three main deposition parameters (Table 3) with two control levels: bath temperature (25 and 50 °C), pulse frequency (0.1 and 1 Hz), and duty cycle (80 and 90%), with the latter defined as follows:

(2) $$Duty\;Cycle\;\left[\%\right]=\frac{t_{ON}}{t_{ON}+t_{OFF}}=\;t_{ON}\cdot f$$

with $t_{ON}$ representing the time in which a cathodic current is applied, $t_{OFF}$ the time in which no current is applied, and f as the pulse frequency in Hz (Figure 1b).

### 2.2. Experimental and Testing

The morphology of the coated samples was investigated using a 3D profilometer (Talysurf CLI 2000, Taylor-Hobson, Leicester, UK) equipped with a surface analysis software (Talymap Universal 3.1.4) collecting the main roughness parameters, roughness average (R_a_) and ten-point height of irregularities (R_z_), according to ISO 4287 [29]. In addition, to macroscopically evaluate the coating morphological features, 3 × 3 mm 3D maps with a 1 µm resolution were collected with the same profilometer. The microscopical morphology of the coatings was studied by means of scanning electron microscope (SEM) micrographs at different magnifications, collected using a 20 kV operating voltage in the secondary electron configuration (SNE ALPHA, SEC Co., Ltd., Suwon, Republic of Korea).

Micro-hardness tests were carried out to assess the mechanical performance of the coatings using depth-sensing micro-indentation (Micro-Combi Tester, CSM Instruments, Peseux, Switzerland). A standard micro-hardness test (micro-Vickers indenter) was performed on the substrate by applying a load of 10 N.

Adhesion resistance is a key parameter to evaluate coating quality. Scratch tests were conducted to determine the adhesion behaviors of each scenario using an 800 µm Rockwell type-A diamond tip (Micro-Combi Tester, CSM Instruments, Switzerland). Tests were conducted in progressive load mode, linearly varying the load from 0.03 to 30 N during the test, imposing a 4 mm scratch path and a 0.2 mm/min speed. Using both the built-in optical microscope and the computation of the change of slope of the tangential force, the normal break load (NBL) was determined, as suggested in [30], to compare the adhesion in different scenarios. In addition, SEM and optical stereoscopic images (HRX-01, Hirox, Tokyo, Japan) were used to evaluate the failure mechanisms of the coatings.

To evaluate the tribological performances of the coated samples a linear reciprocating wear test was conducted using a standard tribometer (CSM Instruments, Needham, MA, USA). The sliding tests lasted 100 m with a normal load of 1 N using a 6 mm diameter 100Cr6 steel ball as a counterpart. All the tests were conducted using a semi-amplitude of 2.5 mm and a speed of 5 cm/s. After each test, by means of a 3D profilometer, the wear volume was calculated. Each wear track was then evaluated using SEM microscopies to determine the different wear mechanisms. In circuit breaker applications, it is useful that the component could efficiently dissipate the heat generated by the current flow to reduce the possible formation of hot spots on the circuit [5]. Thermal performances of the coatings were evaluated through a flash-method test. During the test, the center of the coated surface was heated by a single diode laser pulse (DLR-200-AC, IPG Photonics, Marlborough, MA, USA), with the parameters given in Table 4, while on the opposite surface of specimens, a temperature and infrared (IR) camera with a spectral range of 0.75–14.0 μm (A655S, Teledyne FLIR, Wilsonville, OR, USA) and a thermal sensitivity of 0.05 °C recorded the superficial temperature on the opposite part of the specimens. The thermal diffusivity (*α*) of the samples was computed as follows:

(3) $$\alpha \;\left[\frac{{mm}^{2}}{s}\right]=\frac{d_{sample}^{\;\;\;\;\;\;\;\;\;\;\;\;\;\;\;\;2}}{\tau }$$

where *d_sample_* is the thickness of the specimen in millimeters and *τ* is the heat diffusion time in seconds.

To ensure the applicability of the studied components in the field of electric current transport, electrical resistivity tests were carried out, evaluating possible performance improvements due to the use of graphene as a reinforcement. The resistance of the different scenarios was obtained by recording the electrical resistance of the samples using a resistance meter in a 4-point probe configuration (DC series 2840; B&K Precision, Yorba Linda, CA, USA). Therefore, the resistivity of the components was calculated through the following formula:

(4) $$\rho \;[m\Omega \cdot mm]=\frac{R\cdot A}{l}$$

where ρ is the electrical resistivity, *R* the measured resistance (in milliohm), *A* the cross-section of the sample (in mm^2^), and *l* is the length between the Volt meter probes (in mm).

To properly study the impact of process parameters and their interactions on the main output variables (roughness, NBL, wear volume, and diffusivity), a 3-way ANOVA analysis (Analysis of Variance) was conducted. The analysis was performed using Minitab 21.4 software with a 95% confidence level. In addition, Main Effect Plots (mean response values at each level of the design parameters) are reported and analyzed.

## 3. Results

### 3.1. Morphological Analysis

**Figure 2:**
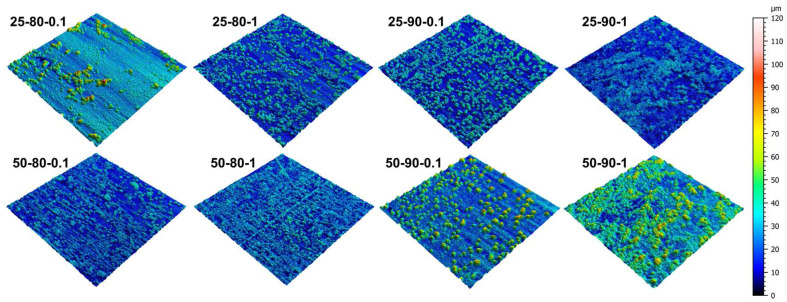
Three-dimensional maps of the coated surfaces.

Figure 2 shows the 3D maps of the composite coatings under varying process parameters. A significant visual difference emerges between the scenarios. Sample 25-80-1 shows a regular surface with localized and isolated protuberances, indicating an uneven deposition of the reinforcement material. On the other hand, an increase in the temperature from 25 to 50 degrees leads to a better distribution of the clusters, which appear more numerous and in a smaller size. It is expected that, with higher temperatures, an increase in the reduction of Cu ions is obtained. This enhancement in the reaction increases the possibility of captured graphene nanosheets into the copper matrix; in fact, a higher presence of copper ions favors the absorption of some of them in GNP flakes, resulting in graphene-based bulges on the surface of composite coatings [31]. This effect can be observed for each value of the duty cycle and the frequency value, suggesting that a better deposition of the composite material is achieved as the bath temperature increases. A change in morphology is also visible as the frequency and duty cycle increase, resulting in surfaces with more distributed clusters and more irregular morphology, due to the greater presence of the reinforcing material. Comparing scenarios varying only the duty cycle, it is observable that the number of bulges in 3D maps increased; this behavior is attributable to the higher available time for the GNPs to be included in the composite coatings with higher duty cycles [32]. Indeed, the 50-90-1 scenario results in the formation of the largest bulge due to the deposition of graphene particles.

**Figure 3:**
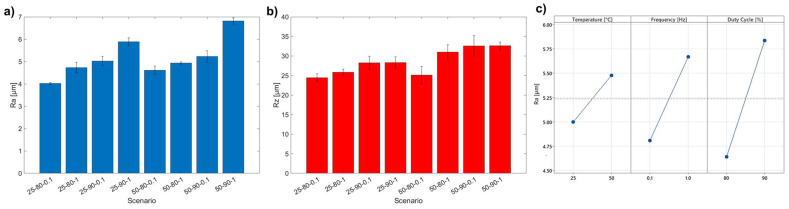
(**a**) R_a_ values; (**b**) R_z_ values; (**c**) Main Effect Plot for R_a_.

The results obtained from the observation of 3D maps are confirmed by the roughness analysis (Figure 3a,b), where the 50-90-1 scenario turns out to be the roughest, with an increase of 70% in R_a_ compared to the 25-80-1 scenario. The visible trend for R_a_ is confirmed by the R_z_ values, whose maximum values recorded show a plateau-like behavior. This is attributable to the adopted deposition process, where highly prominent copper–graphene protuberances tend to collapse during deposition, leveling the surface of the coating [33].

Figure 3c shows the Main Effect Plot for the R_a_ values. It can be seen that the increase in the three parameters studied leads to the increases in the roughness value in a statistically significant way (as shown in ANOVA Analysis subsection). In particular, the increase in roughness is strongly correlated with the increase in the amount of deposited graphene, which acts as a nucleating site for the deposition of copper. This results in a more irregular structure, characterized by several bulges clearly visible in the 3D maps.

**Figure 4:**
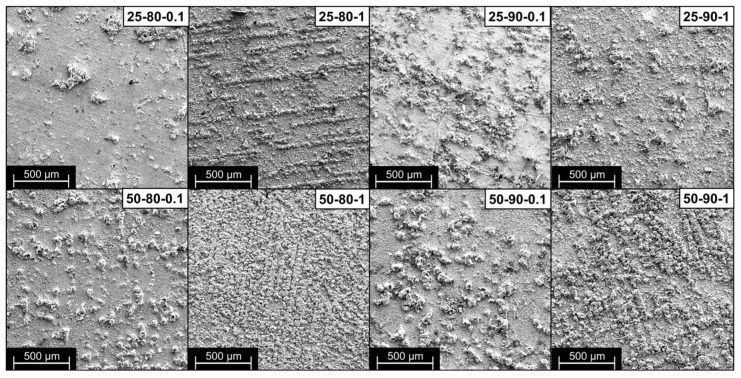
Low-magnification (150×) SEM images of the coatings’ morphology.

Figure 4 shows the low-magnification SEM microscopies of all the studied scenarios. The change in coating morphology as the process parameters are varied is evident and confirms the roughness analysis results. Scenario 25-80-0.1 shows a surface with a largely regular copper deposit with a few distributed bulges due to the local inclusion of GNP flakes. Increasing the duty cycle, frequency, and temperature of the bath leads to an increase in the number of clusters present and to the formation of a more irregular surface but with a high quantity of incorporated graphene, clearly visible in scenarios 25-90-1 and 50-90-1, which, when compared, show the influence of the increase in temperature on the morphology of the material. Higher temperatures favor the inclusion of the reinforcing material in the composite coating, thus favoring the mobility of the particles and ions in the bath and thus achieving greater migration of the particles toward the cathode [34].

**Figure 5:**
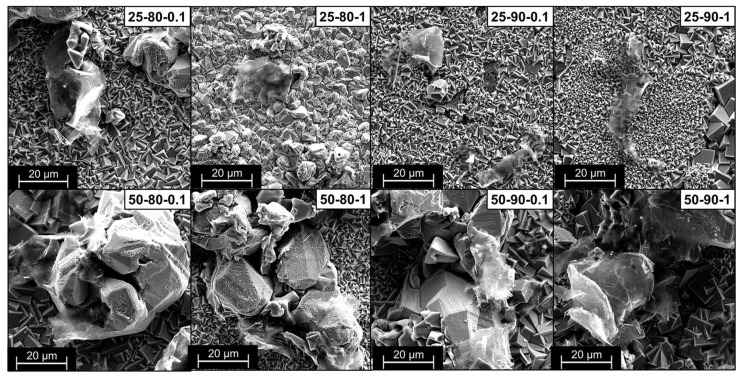
High-magnification (3000×) SEM images of the coatings’ morphology.

SEM microscopies with higher magnification (Figure 5) highlight the presence of graphene flakes and the copper structure that develops embracing them. In addition, it is possible to notice the increase in clusters’ size as the temperature increases and a decrement in the average size of the copper grains for high frequency scenarios, as easily observable in the 25-90-0.1 and 25-90-1 scenarios. This behavior is confirmed by the literature and can be attributed to a different behavior of copper during deposition as the pulse profile changes, going from a grain-growth-guided deposition for low frequencies to a favored nucleation phenomenon in the case of higher frequencies, resulting in smaller grains [35,36].

### 3.2. Microhardness Tests

**Figure 6:**
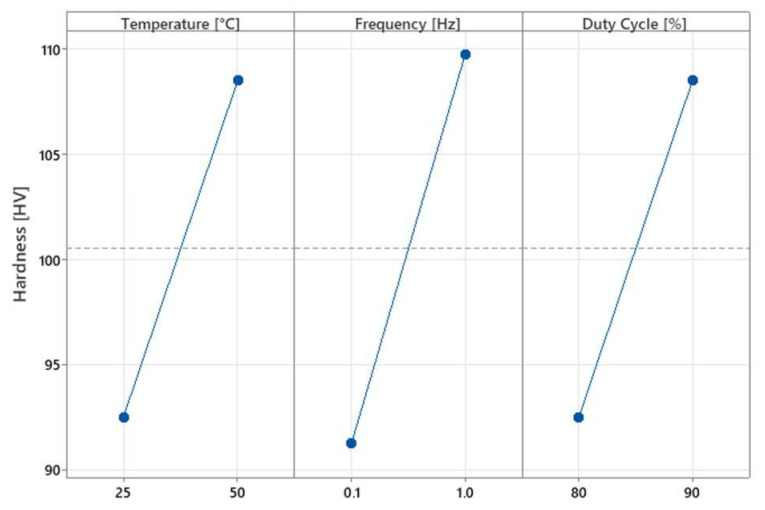
Main Effect Plot for microhardness.

From the hardness data shown in Table 5, it is observed that the presence of electrodeposited copper–graphene coatings confers improved mechanical properties in all scenarios compared to those of bare Cu. Consistent with what was observed for the roughness, the increase in temperature, duty cycle, and frequency value favored the deposition of both copper and graphene in a different extension. Sample 50-90-1 exhibited the highest hardness improvement (76%). From the Main Effect Plots (Figure 6), it is observed that the parameter with the greatest influence on the hardness values is frequency. In fact, this parameter influences the hardness of the samples by acting on two different characteristics: grain size and the amount of graphene deposited. Higher frequencies promoted copper deposition by triggering more nucleation sites, which were generated by applying each pulse. This can be attributed to Hall–Petch hardening, which is achieved by refining the grains of the metal deposit [37]. Simultaneously, increasing the frequency, as demonstrated by [38], promotes the deposition of more reinforcements. Graphene, as reported in the literature [39], is also capable of increasing the hardness of ductile materials within which it is dispersed because of its known specific mechanical properties and the initiation of the Orowan hardening mechanism [40]. Specifically, micrometer particles dispersed in a metal matrix inhibit the prolonged movement of lattice dislocations, representing an obstacle to plastic deformation and eventual crack formation [41]. Also, temperature and duty cycle positively affected the microhardness of the coated samples. This behavior is strongly linked to the Orowan hardening mechanism, as directly influenced by the presence of GNPs. In fact, as previously discussed, temperature and duty cycle increase the GNPs inclusion in the metal matrix and favor the deposition of the GNPs’ migration and inclusion in the coating [31,32].

### 3.3. Tribological Analysis

**Figure 7:**
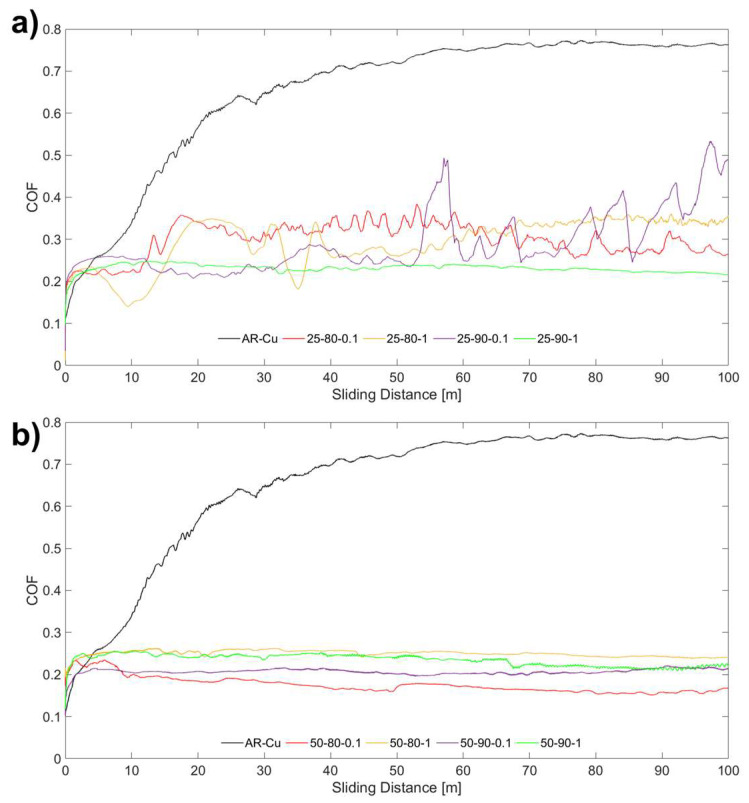
(**a**) Friction curves of 25 °C coated samples; (**b**) friction curves of 50 °C coated samples.

As shown in Figure 7, the introduction of graphene in the coating strongly affects the coefficient of friction compared to the copper substrate, which has a stable friction value of approximately 0.75. It is possible to observe a difference between the COF curves of the scenarios coated at room temperature (Figure 7a) and those at 50 °C (Figure 7b) since the former had a more irregular curve and a higher average friction coefficient. On the other hand, those obtained at a bath temperature of 50 °C exhibited regular behavior, with a friction coefficient that stabilized at values of 0.2 (with a reduction of 66% compared to pure Cu). This trend resulted in different average COF values coherent with the curves in Figure 7, switching from the highest values (0.2989) in scenario 25-80-0.1 to the lowest value (0.1756) in the 50-80-0.1 scenario. It is additionally possible to notice that high-temperature coatings exhibited a smoother friction curve, resulting in lower standard deviation values (reported in Table 6). This can be attributed to the greater inclusion of GNP particles at high bath temperatures. In fact, the many graphene-rich copper agglomerates (Figure 4 and Figure 5) distributed in the coating allow the rapid formation of a homogeneous low-friction layer of graphene at the interface, which is well known for its solid lubricant properties, attributable to its structure [42]. All the values of the friction coefficients turn out to be on the same order of magnitude, precisely, as the properties of graphene, which are not affected by the deposition method.

**Figure 8:**
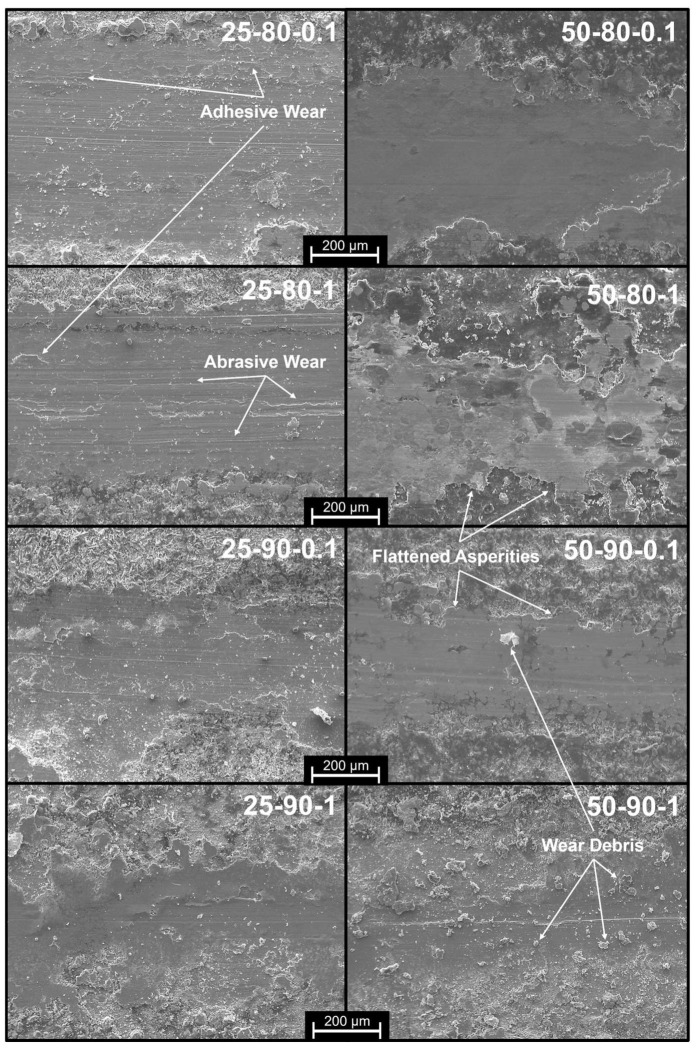
SEM images of the wear tracks.

From the SEM observations (Figure 8) of the samples deposited at 50 °C, it was possible to understand the wear mechanisms of the GNP-lubricated contact. The flattening of asperities turns out to be the dominant mechanism because of the pronounced roughness and the high amount of graphene acting as a lubricant and interlayer between the sample and the counterpart. To support this behavior, the presence of graphene debris on the trace surface is noticeable due to the high amount of reinforcement, which is clearly visible in the 50-90-1 scenario. The presence of adhesive and abrasive wear, which is more common in the case of metallic materials in the absence of lubricant, was not observed. A change in wear mechanisms is observable for samples deposited at room temperature, which is unfavorable for graphene co-deposition. In all scenarios, lines are parallel to the wear groove, as suggested by [43], particularly for low-duty-cycle and low-frequency samples. The presence of abrasive wear can be attributed to a pronounced roughness with an irregular graphene distribution, which does not allow the formation of an effective tribo-film for all the sliding distances. In scenarios such as 25-80-0.1, it is possible to notice the presence of adhesive wear on the edges of the trace, owing to a greater presence of unreinforced copper, which, given its ductility and affinity for its counterpart, generates adhesive wear. In the case of samples obtained under more favorable conditions for the inclusion of graphene (scenario 25-90-1), the presence of the flattering asperity mechanism typical of samples with good graphene content, such as that of samples applied at 50 °C, begins to be observable.

**Figure 9:**
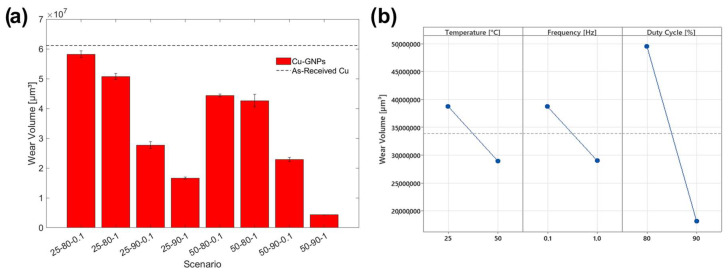
(**a**) Wear volumes of the different scenarios; (**b**) Main Effect Plot of the wear volumes.

The wear volumes, shown in Figure 9a, were lower than those of the as-is copper for each scenario as a result of the reduction in the COF and the increase in hardness due to the inclusion of GNPs. Although the composition of the composite coating changed as the process parameters changed, the friction conditions remained the same in the best scenarios because a graphene tribo-film was formed and detached from the coating. The graphene content in the 50 °C or high-duty cycle and frequency scenarios was sufficient to form a tribo-film between the sample and its counterpart, leading to a significant reduction in wear. It can be observed that the process parameters affect the wear resistance in a manner comparable to that of roughness. In particular, a higher temperature promoted the deposition of a higher fraction of graphene, which improved wear resistance. At low temperatures, copper nucleation is weak, providing fewer nucleating sites that allow the incorporation of graphene into the matrix [31]. From Figure 8 it can be seen that as the temperature changed, with other deposition conditions being equal, a systematic decrease in the volume removed was observed, with a 99 percent improvement in wear resistance in the case of the scenario 50-90-1. A similar behavior was observed by increasing the duty cycle; in fact, higher values favored the inclusion of particle reinforcement. In addition to reducing the friction of the coating, this allows dislocations in the deposited metal to be blocked, promoting hardness and, thus, wear resistance. Increasing the frequency also leads to a benefit for anti-wear properties due to the previously observed increase in hardness, and high frequencies facilitate the reduction in the electric double-layer effect by increasing the deposition of inert particles, generating a greater overpotential that provides more energy for reinforcement absorption [44]. As can be seen in the Main Effect Plot in Figure 9b, the duty cycle parameter is the most relevant for wear resistance, which is strongly related to the amount of reinforcement in the composite coating.

There is an inverse correlation between wear volume and roughness, which confirms the improvement in wear resistance as the amount of graphene deposited increases. As reported in Figure 3a,b, the greater roughness values are attributable to the higher number of graphene flakes embedded in the composite coating. Finally, it is interesting to note that the individual process parameters are found to be statistically significant for wear resistance, as well as the interaction between duty cycle and frequency, as reported in ANOVA Analysis subsection.

### 3.4. Adhesion Tests

**Figure 10:**
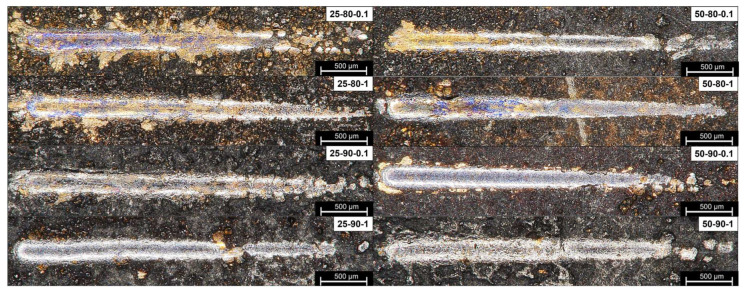
Optical images of the scratch grooves.

**Figure 11:**
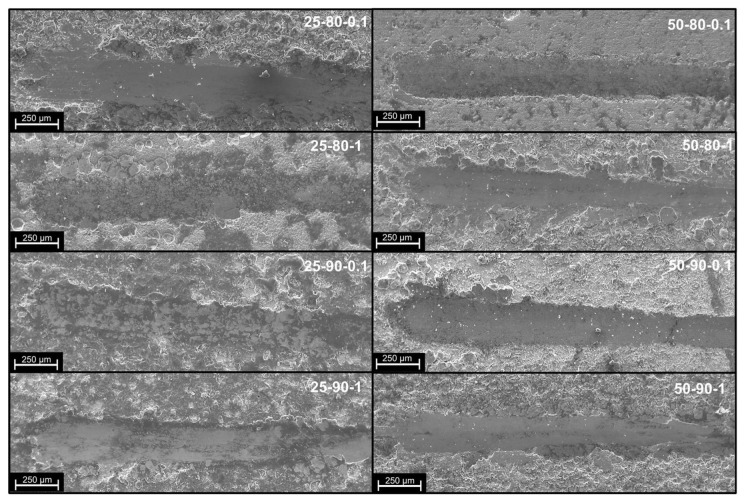
SEM images of the scratch grooves.

**Figure 12:**
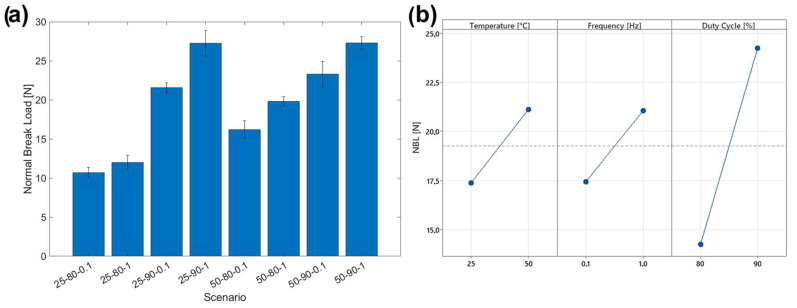
(**a**) NBL of the different coating scenarios; (**b**) Main Effect Plot of NBL.

The adhesion of the coatings to the substrate was tested using scratch testing. The surfaces of the scratch grooves, recorded by SEM and optical microscopy, are shown in Figure 10 and Figure 11. It is possible to identify the mode and mechanism of coating failure, which is mainly plastic deformation. In fact, no brittle or ductile cracks or fractures were observed within the groove, in accordance with the ductile behavior of the copper matrix. It can also be observed, for some scenarios (i.e., 25-80-0.1 and 25-80-1), that the removal of the coating material by plastic deformation both inside and at the borders of the scratch is made visible by the change in surface coloration (Figure 10). In particular, the substrate appears coppery in color, which is much lighter than that of the copper–graphene coating. From the graph of Figure 12a, it is possible to see the correspondence between the NBL values obtained analytically (see Section 2.2) and the values obtained from the observation of the scratch traces shown in Figure 10. It can be observed from the optical images that scenario 25-80-0.1 shows areas inside the scratch, where the coating removal is recorded at much lower loads and for a larger surface area than in the other scenarios. However, for scenarios with higher NBL values (such as 25-90-1 and 50-90-1), the persistence of the coating is observed. The deposited layer is recognizable by the dark color of graphene, which, in its function as a solid lubricant and, at the same time, as a reinforcing filler, opposes the indenter by forming a continuous film. The formation of a continuous film on the groove as well as the exposure of the copper substrate was confirmed by observing the SEM images (Figure 11). In the images shown in (Figure 11), a different shade of coloration (grayscale) is observed, where the lighter area corresponds to the graphene film and the darker area corresponds to the copper substrate. The influence of application parameters on scratch resistance is verified by comparing SEM and optical microscope images (Figure 10 and Figure 11) with NBL values (Figure 12a). Confirming this behavior, a positive trend can be seen as the temperature, duty cycle, and frequency increase (Figure 12b), with sample 50-90-1 reporting a breaking load of approximately 28 N.

### 3.5. Thermal Diffusivity and Electrical Resistivity Evaluation

**Figure 13:**
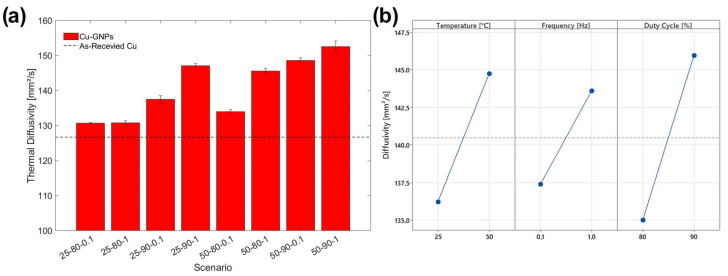
(**a**) Thermal diffusivity values of the samples; (**b**) Main Effect Plot of thermal diffusivity.

The results of the thermal diffusivity tests are shown in Figure 13a. It is evident that the deposition of the copper–graphene coating, even under the most unfavorable conditions for the introduction of large amounts of graphene, improves the thermal diffusivity value by a minimum percentage value of 3.14%, found with the 25-80-0.1 scenario. In fact, as previously reported, low temperature and low duty cycles would lead to fewer graphene inclusions on the composite coatings, reducing the GNP effect in thermal diffusivity. Scenario 25-80-1 showed a comparable diffusivity value; in fact, the only frequency increase did not influence the graphene deposition or, consequently, the thermal properties as much as the other deposition parameters (Figure 13b).

The conditions that favor graphene deposition, i.e., the increase in temperature, frequency, and duty cycle value (see Section 3.1), also led to higher values of thermal diffusivity, as was found with samples 25-90-1, 50-90-0.1, and 50-90-1. The presence of graphene, then, mainly affects the capability of the heat transferring of the sample. Indeed, graphene is known for its heat conduction properties, whether deposited as a coating on a less conductive material or dispersed in a matrix [45]. The planar structure of graphene is responsible for the efficient transmission of heat by mechanical vibrations at the molecular level [46]. In composite materials, graphene flakes, even if they are not organized in a continuous structure, represent local short tracks that facilitate heat dissipation on the plane [47]. Main Effect Plots evidence that the duty cycle value has the largest influence on the efficacy of graphene deposition (Figure 13b), as already verified in roughness analysis (see Figure 3c). The improvement obtained in thermal diffusivity plays an important role in power breaker applications, avoiding the formation of hot spots that could lead to damage to the electrical components or reductions in efficiency.

The deposition parameters similarly affect the resistivity, positively influenced by the presence of graphene. It is observed from the values shown in Table 7 that the resistivity values of the samples deposited with copper–graphene are lower than those of the bare copper in all scenarios. The 25-80-0.1 scenario is confirmed as the specimen with the smallest reduction, about 2.26%, while 50-90-1 shows a 15.7% resistivity decrement. It can be seen, from Table 7, that the increase in temperature leads to an improvement in electrical conductivity, attributable, therefore, to a greater amount of graphene dispersed more evenly. The same considerations can be highlighted for increases of the duty cycle, favoring the co-deposition of Cu-GNPs. Similarly, an increase in frequency leads to a lower reduction in electrical resistivity; in fact, higher frequency allows a greater graphene presence while reducing grain size, notoriously penalizing electrical conductivity [48].

### 3.6. ANOVA Analysis

Table 8 shows the *p*-values of the three-way ANOVA analysis, conducted by varying the process parameters. What is evident is the statistical significance of the individual process variables with respect to the main experimental outputs. In fact, it is observable that the *p*-value is always less than 0.05 for each control variable.

In contrast, the synergistic and anti-synergistic effects of parameter pairs are not always found to be statistically significant. In particular, the most significant interaction appears to be that between the duty cycle and the frequency (with a *p*-value less than 0.05 for R_a_, NBL, and wear volumes). For all output variables, the analysis appears to be particularly reliable, as can be seen from the value of the R-squared parameter, which is always above 70 percent.

## 4. Conclusions

In conclusion, the study demonstrates that the process parameters—bath temperature, duty cycle, and frequency—substantially influence the morphology, roughness, hardness, wear resistance, and thermal diffusivity of copper–graphene composite coatings.

- Increasing the bath temperature enhances the distribution and incorporation of GNPs within the coating, resulting in an irregular morphology with higher GNP presence, as evidenced by 3D mapping and roughness data.
- Higher duty cycles and frequencies also promote the deposition of graphene, leading to a more pronounced roughness and improved hardness, as the graphene particles act as nucleation sites, facilitating Hall–Petch and Orowan hardening mechanisms, obtaining a 76% maximum hardness improvement.
- SEM and friction analyses reveal that high-temperature scenarios, such as 50-90-1, foster the formation of a stable, tribo-film of graphene, which significantly reduces wear and the coefficient of friction. This scenario also exhibited a remarkable 99% improvement in wear resistance, underscoring the tribological benefits of enhanced graphene deposition.
- Scratch testing results confirmed the strong adhesion of the coatings, with higher parameter values leading to more resilient layers that resist plastic deformation and form a continuous graphene film during testing. This behavior, coupled with the improved thermal diffusivity observed in graphene-rich coatings (more than 19% improvement), highlights copper–graphene coatings as efficient heat conductors for electronics.

Ultimately, the findings indicate that optimizing deposition parameters can maximize the mechanical and thermal performance of copper–graphene composite coatings. Therefore, the application of optimized Cu-GNP-coated circuit breakers will be exploited to test the behaviors in real-life scenarios and evaluate the environmental and economic benefits of industry-scale manufacturing.

## Figures and Tables

**Table 1:** GNPs technical datasheet.

Property	Value	Unit
Carbon Content	>98	%
C:O Ratio	45:1	-
Specific Surface Area	30	M^2^/g
Thickness	14	nm
Avg. Particle Size D90	15	µm
Avg. Particle Size D50	10	µm

**Table 2:** Electroplating bath composition.

Reagent	Concentration	Unit
CuSO_4_	170	g/L
CuCl_2_	50	p.p.m.
GNPs	0.8	g/L

**Table 3:** Full factorial plan obtained varying bath temperature, duty cycle, and frequency.

Scenario	Temperature [°C]	Duty Cycle [%]	Frequency [Hz]
25-80-0.1	25	80	0.1
25-80-1	25	80	1
25-90-0.1	25	90	0.1
25-90-1	25	90	1
50-80-0.1	50	80	0.1
50-80-1	50	80	1
50-90-0.1	50	90	0.1
50-90-1	50	90	1

**Table 4:** Laser’s main features.

Features	Value	Unit
Emission centroid wavelength	975 ± 5	nm
Emission Line-width	6	nm
Maximum power	200	W
Beam Parameter Product	22	mm·mrad
Output fiber core diameter	200	μm
Output beam diameter	5	mm

**Table 5:** Microhardness test results.

Scenario	Avg. Value [HV]	St. Dev.
AR-Cu	72	0.8
25-80-0.1	78	1.3
25-80-1	97	1.5
25-90-0.1	89	2.1
25-90-1	106	2.4
50-80-0.1	86	1.6
50-80-1	109	1.4
50-90-0.1	112	2.5
50-90-1	127	2.1

**Table 6:** Average COF and standard deviation of COF curves.

Scenario	Average COF	St. Dev. of COF Curves
AR-Cu	0.6518	0.1627
25-80-0.1	0.2989	0.0399
25-80-1	0.2938	0.0564
25-90-0.1	0.2895	0.0722
25-90-1	0.2312	0.0135
50-80-0.1	0.1756	0.0189
50-80-1	0.2500	0.0075
50-90-0.1	0.2068	0.0067
50-90-1	0.2348	0.0095

**Table 7:** Electrical resistivity of the coated samples compared to the AR copper.

Scenario	Resistivity mΩ·mm	St. Dev.
AR-Cu	1677	10
25-80-0.1	1639	15
25-80-1	1545	23
25-90-0.1	1601	42
25-90-1	1428	9
50-80-0.1	1494	12
50-80-1	1430	27
50-90-0.1	1451	34
50-90-1	1414	18

**Table 8:** ANOVA results for the main outputs.

	Ra [µm]		Hardness [HV]		NBL [N]		Wear Volumes [µm³]		Diffusivity [mm²/s]	
Source	F-Value	*p*-Value	F-Value	*p*-Value	F-Value	*p*-Value	F-Value	*p*-Value	F-Value	*p*-Value
**Temperature [°C]**	16.46	**0.003**	31.54	**0.041**	64.36	**0.000**	64.44	**0.000**	31.76	**0.000**
**Duty Cycle [%]**	101.82	**0.000**	48.11	**0.038**	459.77	**0.000**	660.5	**0.000**	52.49	**0.000**
**Frequency [Hz]**	52.51	**0.000**	23.78	**0.045**	60.91	**0.000**	62.95	**0.000**	16.94	**0.003**
**Temperature [°C]** **×** **Duty Cycle [%]**	0.61	0.455	10.03	0.382	38.36	**0.000**	0.8	0.395	0.07	0.795
**Temperature [°C] × Frequency [Hz]**	0.08	0.784	7.51	0.453	1.1	0.321	0.03	0.874	0.79	0.397
**Duty Cycle [%] × Frequency [Hz]**	7.63	**0.022**	0.45	0.746	6.11	**0.035**	20.14	**0.002**	0.11	0.748
**R-Squared [%]**	92.03		72.21		97.65		96.56		86.51	

## Data Availability

The original contributions presented in this study are included in the article. Further inquiries can be directed to the corresponding author.
